# Takinib inhibits microglial M1 polarization and oxidative damage after subarachnoid hemorrhage by targeting TAK1-dependent NLRP3 inflammasome signaling pathway

**DOI:** 10.3389/fimmu.2023.1266315

**Published:** 2023-11-14

**Authors:** Weihan Wang, Cong Pang, Jiaxing Zhang, Lei Peng, Xianghua Zhang, Lin Shi, Hao Zhang

**Affiliations:** ^1^ Department of Neurosurgery, Beijing Friendship Hospital, Capital Medical University, Beijing, China; ^2^ Department of Neurosurgery, The Affiliated Huai’an No.1 People’s Hospital of Nanjing Medical University, Huai’an, China; ^3^ Graduate School of Capital Medical University, Beijing, China

**Keywords:** subarachnoid hemorrhage, early brain injury, takinib, TAK1, NLRP3

## Abstract

Transforming growth factor-β-activated kinase 1 (TAK1) positively regulates oxidative stress and inflammation in different diseases. Takinib, a novel and specific TAK1 inhibitor, has beneficial effects in a variety of disorders. However, the effects of takinib on early brain injury (EBI) after subarachnoid hemorrhage (SAH) and the underlying molecular mechanisms remain unknown. Our study showed that takinib administration significantly inhibited phosphorylated TAK1 expression after SAH. In addition, takinib suppressed M1 microglial polarization and promoted M2 microglial polarization. Furthermore, blockade of TAK1 by takinib reduced neuroinflammation, oxidative damage, brain edema, and neuronal apoptosis, and improved neurological behavior after SAH. Mechanistically, we revealed that TAK1 inhibition by takinib mitigated reactive oxygen species (ROS) production and ROS-mediated nod-like receptor pyrin domain-containing protein 3 (NLRP3) inflammasome activation. In contrast, NLRP3 activation by nigericin abated the neuroprotective effects of takinib against EBI after SAH. In general, our study demonstrated that takinib could protect against EBI by targeting TAK1-ROS-NLRP3 inflammasome signaling. Inhibition of TAK1 might be a promising option in the management of SAH.

## 1 Introduction

Subarachnoid hemorrhage (SAH) remains a life-threatening disease with poor prognosis. How to improve the clinical outcome after SAH is still a tough challenge. Multiple pathophysiological processes occurring after SAH, including early brain injury (EBI), cerebral vasospasm, and delayed cerebral ischaemia (1). Till now, mounting evidence has demonstrated that the neurological outcome after SAH is seriously influenced by early brain injury (EBI) (2–4). Multiple cellular mechanisms are involved in the EBI pathophysiology after SAH. Among them, activation of inflammatory response and oxidative damage contribute greatly to the development of EBI (5–7). Thus, a potential therapy for improving the prognosis after SAH is intervening neuroinflammation and oxidative stress.

Transforming growth factor-β-activated kinase 1 (TAK1), a member of the mitogen‐activated protein kinase (MAPK) family, has a wide range of biological functions (8–10). It has demonstrated that TAK1 activation could strongly elicit pro-inflammatory cytokines release and drive microglial toward a proinflammatory phenotype (11–14). In addition, TAK1 mediates reactive oxygen species (ROS) generation to aggravate oxidative damage in different diseases models (15, 16). In central nervous system (CNS), TAK1 is mainly expressed in neurons and could regulate a variety of intracellular signaling pathways, including nuclear factor‐κB (NF‐κB), AMP-activated protein kinase (AMPK), and nod-like receptor pyrin domain-containing protein 3 (NLRP3) inflammasome (17–19). Since these molecular mechanisms contribute to EBI pathophysiology, targeting TAK1 might be an effective treatment for SAH.

Takinib, a novel and specific TAK1 inhibitor, has been recently evaluated in a variety of disease models (20–22). Previous studies have demonstrated that takinib could inhibit inflammation and apoptosis by suppression of TAK1-mediated signaling pathway (20, 21). However, whether takinib could protect against EBI after SAH and the potential molecular mechanisms remain unclear. Hence, this study aimed to investigate the possible role of takinib in EBI after SAH and its underlying mechanisms.

## 2 Materials and methods

All the experimental procedures were approved by the Animal Ethics Review Committee of Beijing Friendship Hospital and carried out in accordance with the National Institutes of Health guidelines. A total of 190 rats (weighing 250 to 300 g) were used in our study. Among them, 154 rats underwent SAH insults and 8 rats were excluded due to mild SAH grading score (less than 8) and intracerebral hematoma. All rats were numbered consecutively and randomization was conducted by using the website Randomization (http://www.randomization.com).

### 2.1 SAH model

The SAH model was performed according to our previous studies (23, 24). Briefly, rats were anesthetized with avertin (200 mg/kg). After anesthetization, rats were operated in a stereotactic frame. A hole was drilled into the skull in the midline 7.5 mm anterior to the bregma. SAH animals were injected with 0.35 ml of nonheparinized fresh autologous arterial blood from the femoral artery through the burr hole under aseptic conditions. Sham group rats underwent the same procedures, but were injected with 0.35 ml physiological saline. The basal temporal lobe adjacent to the clotted blood were collected for evaluation. After recovering from anesthesia, rats were housed in their cages and were free to food and water.

### 2.2 Experiment design

In the first set of experiments, the dose-dependent effects of takinib on TAK1 activation were examined. Total of 70 rats were randomly assigned into 5 groups, including sham + vehicle (n = 12), SAH + vehicle (n = 15, 3 rats died), SAH + 0.1 mM takinib (n = 15, 3 rats died), SAH + 0.3 mM takinib (n = 14, 2 rats died), and SAH + 0.9 mM takinib (n = 14, 2 rats died). Western blotting and double immunofluorescence staining were conducted to show the protein expression and cellular distribution of TAK1 in the brain cortex.

In the second set of experiments, we further explored the effects of takinib on microglial activation, ROS overproduction, and the subsequent brain damage after SAH. A total of 83 rats were randomly assigned into 4 groups, including sham + vehicle (n = 18), SAH + vehicle (n = 22, 4 rats died), SAH + 0.9 mM takinib (n = 21, 3 rats died), and SAH + 0.9 mM takinib + nigericin (n = 22, 4 rats died). Western blotting, double immunofluorescence staining, biochemical estimation, brain edema, and neurological behavior were conducted to determine the effects of takinib on EBI after SAH and the possible mechanisms.

In the third set of experiments, we investigated the effects of takinib on histopathological change and neurological behavior at day 3 after SAH. A total of 29 rats were randomly assigned into 4 groups, including sham + vehicle (n = 6), SAH + vehicle (n = 8, 1 rats died), SAH + 0.9 mM takinib (n = 7, 1 rats died), and SAH + 0.9 mM takinib + nigericin (n = 8, 1 rats died). Nissl staining and neurological behavior were performed. The experiment design and timeline were shown in **Supplementary Figure 1**.

### 2.3 Drug administration

Takinib (Selleck) was dissolved in dimethyl sulfoxide (DMSO) and diluted in physiologic saline at concentrations of 0.1, 0.3, and 0.9 mM. Various doses of takinib (10 μl) or vehicle was administered into the left lateral ventricle at 30 min post SAH insults. Nigericin (Selleck, 2 μg) was dissolved in 2 μl ethanol and physiologic saline and was administered to rats by intracerebroventricular route 2 h before SAH construction. For the intracerebroventricular administration, rats were put on a stereotactic frame. Coordinates for the intracerebroventricular injection were 1.5 mm posterior and 1.0 mm lateral to bregma, and 4.5 mm below the dural layer. The doses of takinib and nigericin were selected according to previous studies (20, 25).

### 2.4 Neurological scoring

Neurological function was evaluated by using a neurological severity scoring system as previously reported (26). Six test sub-scores were included in this scoring system. The high scores represented a relative normal neurological function. In addition, the rotarod test was conducted to measure motor function. The duration on the rotarod was recorded for statistical analysis (1, 27).

### 2.5 Brain water content

Brain water content was determined by using a wet/dry method (28). At 24 h following SAH, rats were deeply anesthetized with an overdose of avertin and the brains were separated into three parts, including cerebrum, cerebellum and brain stem. The wet weight was recorded. Each part of brain was dried at 80°C for 72 h and weighed again (dry weight). Brain water content was calculated by [(wet weight − dry weight)/wet weight] × 100%.

### 2.6 Western blotting

Western blotting was conducted according to previous studies (23, 29). In brief, the protein samples were separated by SDS-PAGE gel. And then they were transferred to nitrocellulose membrane. Afterward, the membranes were incubated with primary antibodies against TAK1 (1:1000, Cell Signaling), p-TAK1 (1:1000, Cell Signaling), NLRP3 (1:200, Santa Cruz Biotechnology), ASC (1:200, Santa Cruz Biotechnology), caspase-1 (1:200, Santa Cruz Biotechnology), cleaved caspase-1 (1:200, Santa Cruz Biotechnology), 3-nitrotyrosine (1:2000, Abcam), and β-actin (1:3000, Bioworld Technology) overnight at 4°C. After that, appropriate secondary antibodies were incubated at room temperature. Membranes were then exposed by ECL reagent.

### 2.7 Immunofluorescence

Immunofluorescence staining was conducted according to previous studies (23, 24). In brief, brain sections were blocked with 5% donkey serum. And then they were incubated overnight at 4°C with primary antibodies against p-TAK1 (1:100, Cell Signaling), CD16/32 (1:100, BD Biosciences), CD206 (1:100, Invitrogen), NLRP3 (1:50, Santa Cruz Biotechnology), 8-hydroxydeoxyguanosine (8-OhdG) (1:100, Abcam), IL-1β (1:100, Abcam), NeuN (1:200, EMD Millipore), and Iba-1 (1:50, Santa Cruz Biotechnology). Next, the brain sections were incubated with appropriate secondary antibodies. The fluorescently stained cells were visualized and photographed under a fluorescence microscope.

### 2.8 TUNEL staining

TUNEL staining (Beyotime) was conducted in line with the operation instructions. In brief, the brain sections were incubated with primary antibody against NeuN overnight. Afterward, the slides were incubated with TUNEL reaction mixture. Sections were visualized and photographed under a fluorescence microscope.

### 2.9 Malondialdehyde detection

The level of malondialdehyde (MDA) in brain samples were examined according to the manufacturer’s instructions (Nanjing Jiancheng Bioengineering Institute). MDA was determined at the wavelength of 535 nm using a spectrophotometer.

### 2.10 Statistical analysis

All values were expressed as means ± s.d. GraphPad Prism 8.02 was used to conduct statistical analysis. All data were tested for normality via Shapiro–Wilk test. Measurements were analyzed with one-way ANOVA followed by Tukey’s *post-hoc* test. *P <*0.05 was verified as statistically different.

## 3 Results

### 3.1 Effects of takinib on TAK1 activation after SAH

**Figure 1:**
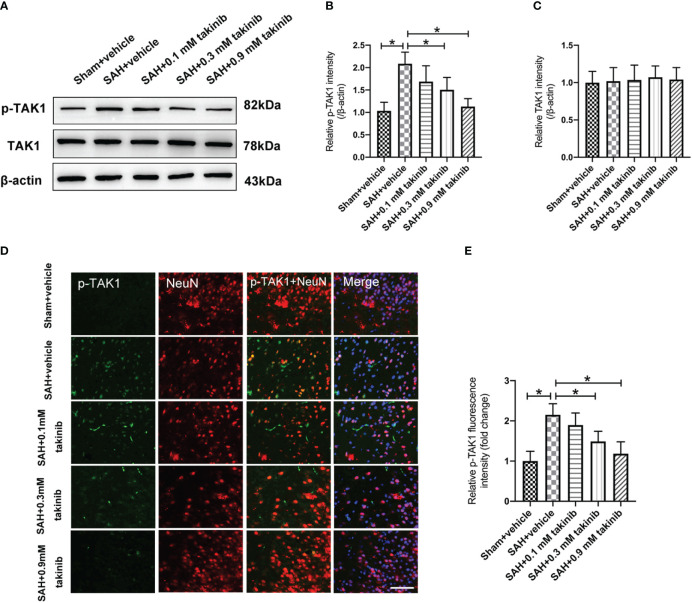
Dose-response effects of takinib on TAK1 activation after SAH. **(A)** Representative western blot bands and quantitative analyses of p-TAK1 **(B)** and TAK1 **(C)** after SAH (n = 6 each group). **(D)** Representative photomicrographs and quantification **(E)** of p-TAK1 immunofluorescence staining in the basal cortex at 24 h after SAH (n = 6 each group). One-way ANOVA with Tukey, bars represent the mean ± SD. ^*^ *P* < 0.05.

Takinib is a novel and highly selective TAK1 inhibitor. However, the influence of takinib on TAK1 activation after SAH remains obscure. Western blotting was conducted to detect the expression of p-TAK1 and TAK-1 after SAH. The results showed that doses of 0.3 mM and 0.9 mM takinib, but not 0.1 mM takinib, markedly inhibited p-TAK1 expression as compared with SAH + vehicle group (**Figures 1A, B**). There was no significant difference among all experimental groups in total TAK1 expression (**Figure 1C**). It has been demonstrated that TAK1 is mainly expressed in neurons. Consistent with previous studies, double immunofluorescence revealed that TAK1 activation was mainly distributed in neurons after SAH. In contrast, takinib treatment at 0.3 mM and 0.9 mM could significantly suppress TAK1 activation in neurons (**Figures 1D, E**). Since 0.9 mM takinib had the maximal effects, we used this dose in the subsequent experiments.

### 3.2 Influence of takinib on NLRP3 inflammasome signaling after SAH

**Figure 2:**
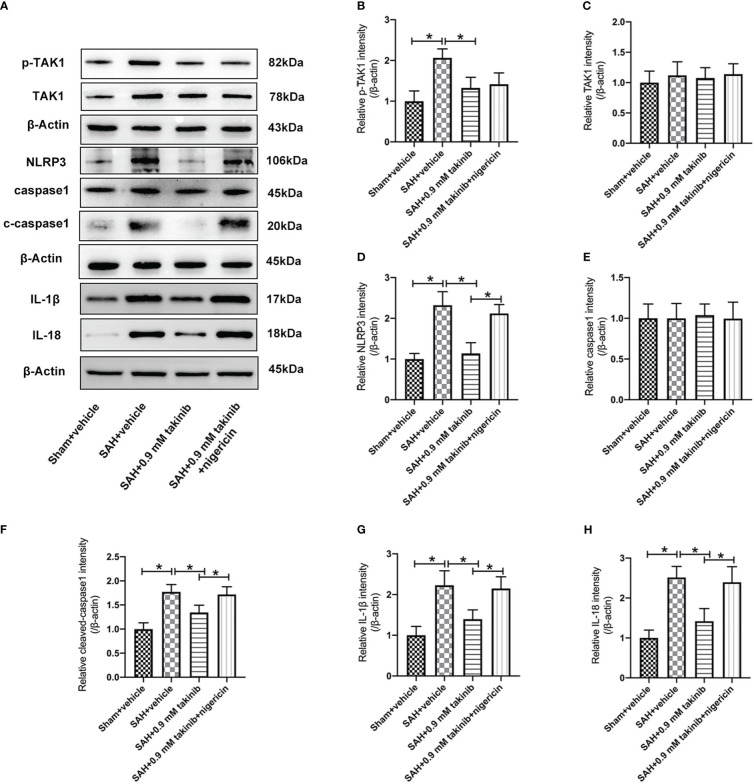
Effects of takinib on TAK1-NLRP3 inflammasome activation after SAH. **(A)** Representative western blot bands and quantitative analyses of p-TAK1 **(B)**, TAK1 **(C)**, NLRP3 **(D)**, caspase1 **(E)**, cleaved caspase1 **(F)**, IL-1β **(G)**, and IL-18 **(H)** after SAH (n = 6 each group). One-way ANOVA with Tukey, bars represent the mean ± SD. ^*^ *P* < 0.05.

TAK1 has been verified as a key regulator of NLRP3 inflammasome activation. However, whether takinib could modulate NLRP3 inflammasome signaling after SAH remains unknown. In this experiment, we evaluated the effects of takinib on NLRP3 inflammasome signaling after SAH. As shown, western blotting data revealed that SAH insults significantly induced the protein levels of p-TAK1, NLRP3, ASC, cleaved caspase1, IL-1β, and IL-18, all of which were reversed by takinib treatment (**Figures 2A–H**). However, in addition to p-TAK1, other molecular targets alterations by takinib were counteracted by nigericin administration (**Figures 2A–H**). No significant differences in the expressions of TAK1 (**Figure 2C**) and caspase1 (**Figure 2E**) were detected among all experimental groups.

### 3.3 Influence of takinib on M1/M2 microglial polarization after SAH

**Figure 3:**
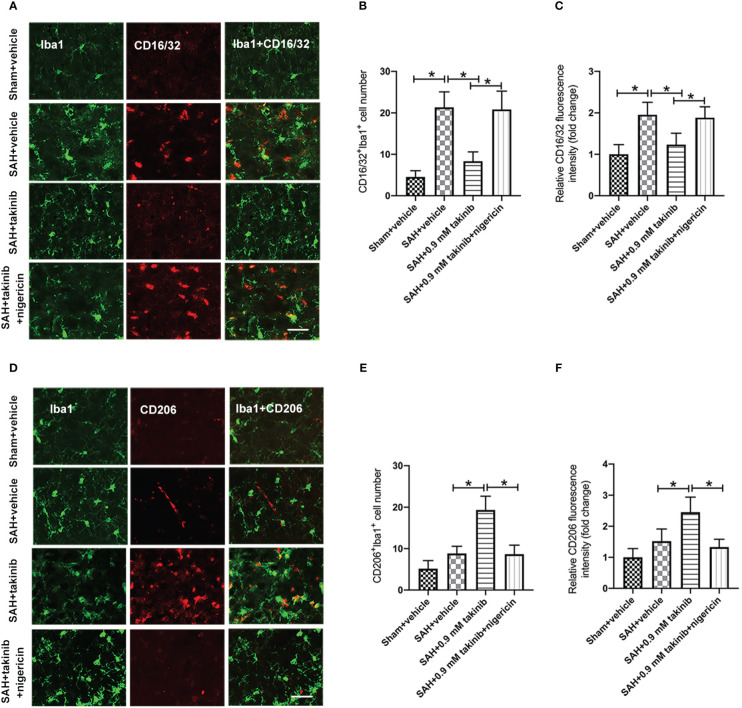
Effects of takinib on microglial phenotypic transformation after SAH. **(A)** Double immunofluorescence staining for CD16/32 in microglial in the basal cortex after SAH. **(B, C)** Quantification of CD16/32 immunofluorescence staining in the basal cortex at 24 h after SAH (n = 6 each group). **(D)** Double immunofluorescence staining for CD206 in microglial in the basal cortex after SAH. **(E, F)** Quantification of CD206 immunofluorescence staining in the basal cortex at 24 h after SAH (n = 6 each group). One-way ANOVA with Tukey, bars represent the mean ± SD. ^*^ *P* < 0.05.

Microglial polarization plays a critical role in neuroinflammation after SAH. Numerous studies have demonstrated that TAK1 could induce microglial activation. However, whether TAK1 could modulate microglial polarization after SAH remains elusive. We then evaluated the effects of takinib on M1/M2 microglial polarization after SAH. The double immunofluorescence showed that takinib treatment significantly reduced the proportion of M1 microglial (CD16/32^+^) and enhanced the quantity of M2 microglial (CD206^+^) following SAH (**Figures 3A–F**). In contrast, NLRP3 activator nigericin abated the effects of takinib on M1/M2 microglial polarization (**Figures 3A–F**).

### 3.4 Influence of takinib on ROS production, and oxidative damage after SAH

**Figure 4:**
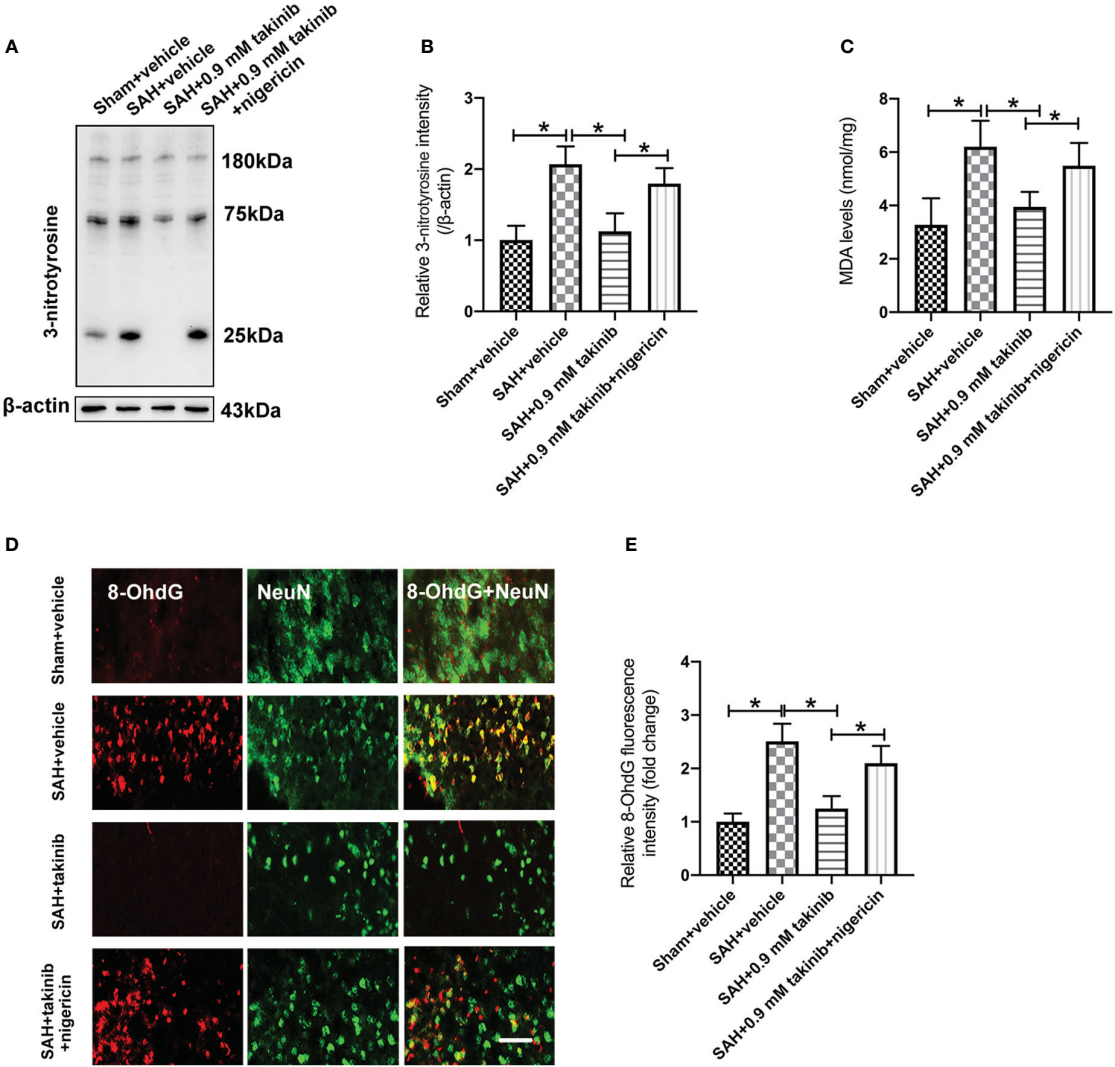
Effects of takinib on ROS and oxidative damage after SAH. Western blot assay **(A)** and quantification **(B)** for expression of 3-nitrotyrosine in different groups (n = 6 each group). **(C)** Quantification of MDA level in brain tissue at 24 h post-SAH (n = 6 each group). **(D)** Representative photomicrographs and quantification **(E)** of 8-OhdG immunofluorescence staining in the basal cortex after SAH (n = 6 each group). One-way ANOVA with Tukey, bars represent the mean ± SD. ^*^ *P* < 0.05.

ROS overproduction plays a key role in oxidative damage and contributes greatly to the development of EBI after SAH. Previous studies have demonstrated that TAK1 activation could aggravate ROS overproduction and oxidative damage. However, the influence of takinib on ROS production and oxidative damage following SAH remains unknown. We further evaluated the effects of takinib on ROS production and oxidative damage after SAH. 3-nitrotyrosine, a major product of tyrosine oxidation, is an important biomarker for ROS production. MDA is a biological marker for oxidative damage and lipid peroxidation. Our data revealed that SAH insults significantly induced ROS overproduction, lipid peroxidation, and oxidative damage, all of which were ameliorated by takinib treatment (**Figures 4A–E**). In contrast, the anti-oxidative effects of takinib could be partly reversed by nigericin treatment (**Figures 4A–E**).

### 3.5 Influence of takinib on neurological function, brain edema, and neuronal apoptosis at day 1 after SAH

**Figure 5:**
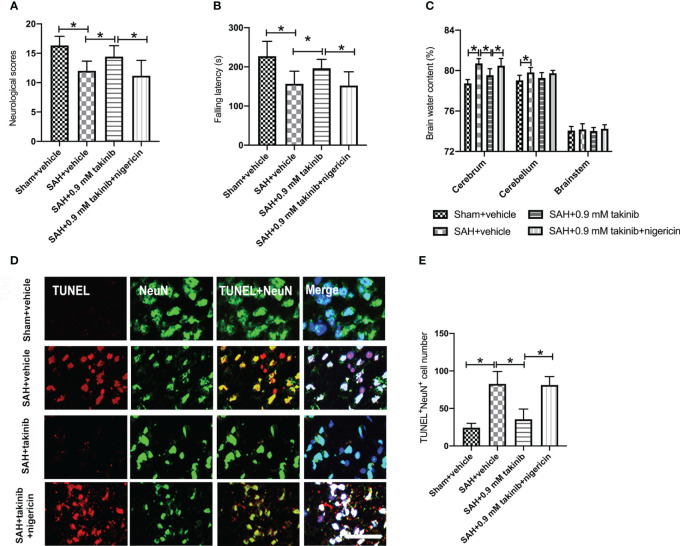
Effects of takinib on neurological behavior, brain water content, and neuronal survival after SAH. Quantification of **(A)** neurological deficits scores, **(B)** rotarod performance, and **(C)** brain water content in different groups after SAH (n = 10-12 each group). **(D)** Representative photomicrographs and quantification **(E)** of TUNEL staining in the basal cortex after SAH (n = 6 each group). One-way ANOVA with Tukey, bars represent the mean ± SD. ^*^ *P* < 0.05.

We then evaluated the effects of takinib on neurological outcomes, brain edema, and neuronal apoptosis after SAH. Consistent with the reduced neuroinflammation and oxidative damage, takinib treatment significantly improved neurological scores and motor functions, ameliorated brain edema, and reduced neuronal apoptosis after SAH (**Figures 5A–E**). In contrast, the pretreatment of nigericin statistically deteriorated the beneficial effects of takinib on neurological behavior, brain edema, and neuronal apoptosis after SAH (**Figures 5A–E**).

### 3.6 Influence of takinib on histopathological change and neurological behavior at day 3 after SAH

**Figure 6:**
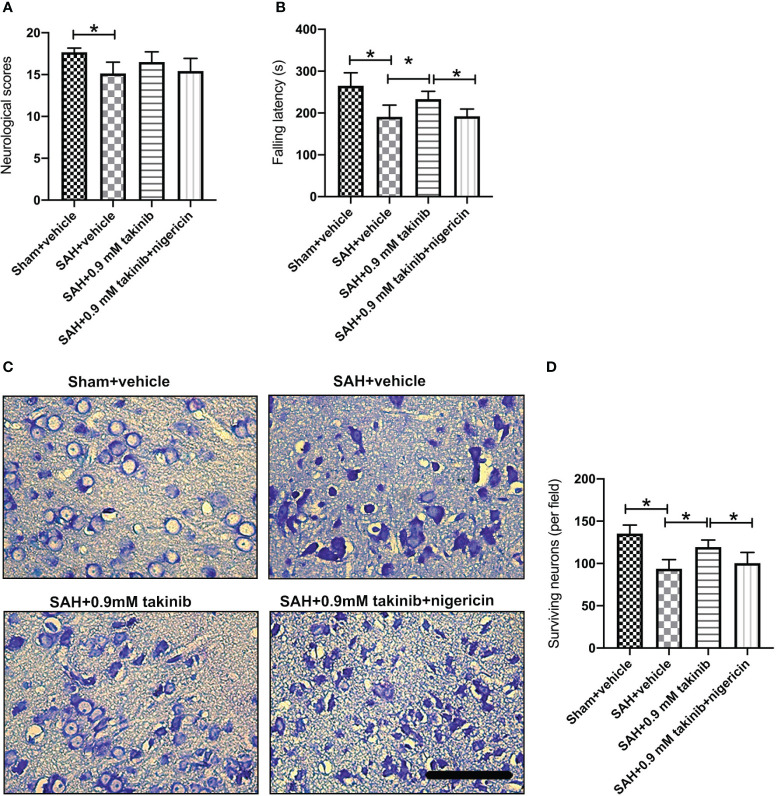
Effects of takinib on histopathological change and neurological behavior at day 3 after SAH. Quantification of **(A)** neurological deficits scores and **(B)** rotarod performance in different groups after SAH (n = 6 - 7 each group). **(C)** Representative photomicrographs and quantification **(D)** of survival neurons in the basal cortex after SAH (n = 6 each group). One-way ANOVA with Tukey, bars represent the mean ± SD. ^*^ *P* < 0.05.

The first 72 h following SAH play a vital role in determining overall outcome. We then investigated the effects of takinib on histopathological change and neurological behavior at day 3 after SAH. It indicated that SAH insults induced an evident histopathological damage in brain cortex as evidenced by sparse cell arrangements and integrity loss. In contrast, takinib significantly improved histopathological change and reduced neuronal degeneration. In addition, takinib also improved motor function in the early period after SAH. However, all these cerebroprotective effects were abated by nigericin (**Figures 6A–D**).

## 4 Discussion

**Figure 7:**
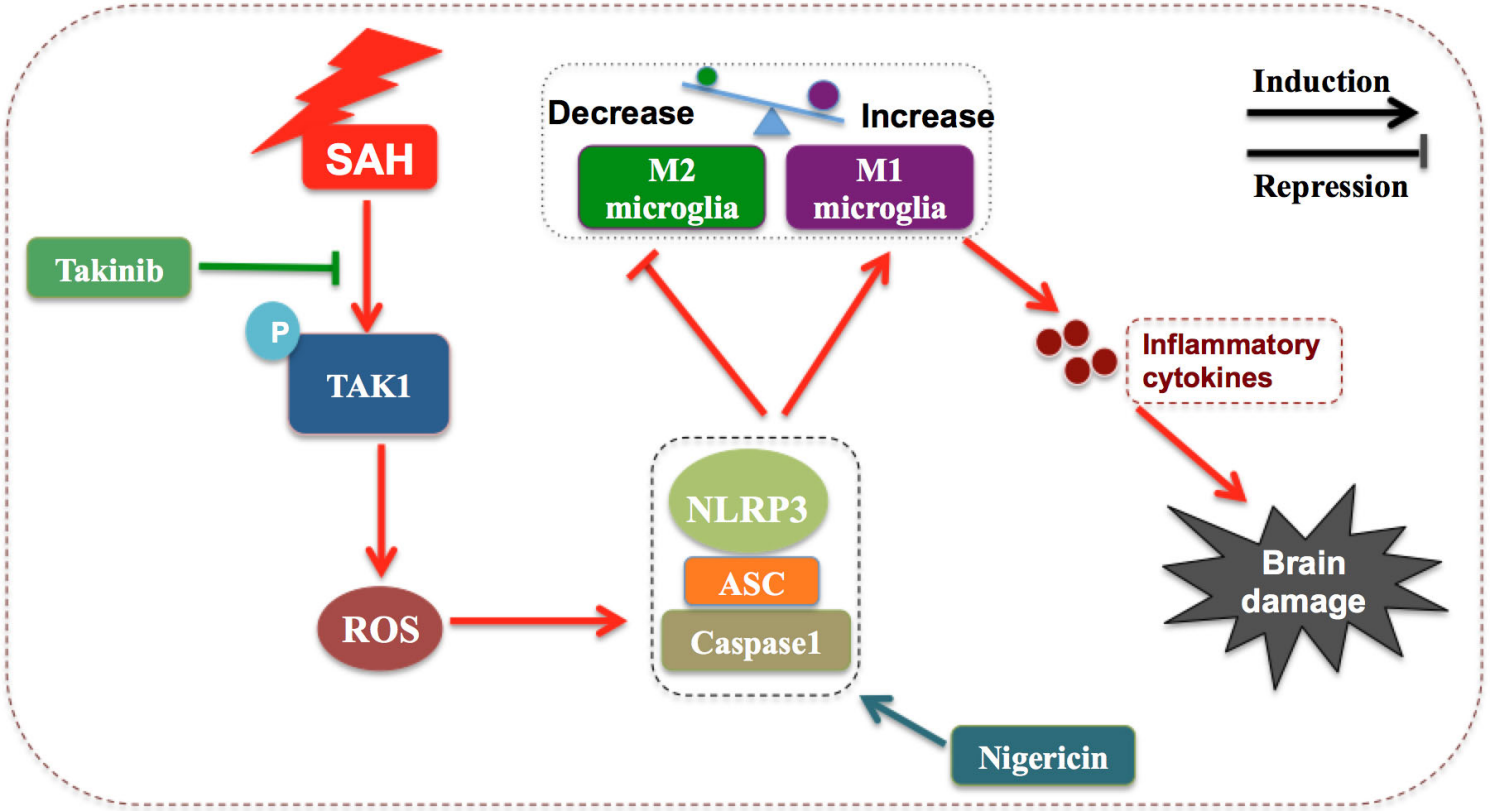
Schematic illustrating the possible mechanisms of takinib action after SAH. As illustrated, SAH significantly increases the expression level of p-TAK1(Thr187) indicating that TAK1 is activated after SAH. TAK1 activation triggers reactive oxygen species (ROS) generation. In response to ROS accumulation after SAH, NLRP3 recruits the adaptor apoptosis-related speck-like protein (ASC) and pro-caspase-1 to form a large multiprotein complex. NLRP3 inflammasome activation promotes microglial phenotype toward M1 and inhibits M2 microglial polarization after SAH, subsequently aggravating neuroinflammation and brain damage. Takinib, a novel and highly selective TAK1 inhibitor, could suppress TAK1 activation and TAK1-mediaed ROS-NLRP3 inflammasome signaling after SAH. In contrast, NLRP3 inflammasome activator nigericin reverses the beneficial effects of takinib against SAH, eventually aggravating SAH-induced brain damage.

In this study, we verified the beneficial effects of takinib on EBI after SAH. Our data indicated that takinib could ameliorate SAH-induced inflammatory response by inhibiting M1-microglial phenotype polarization and promoting microglial polarization to M2 phenotype. In addition, takinib reduced ROS generation and suppressed oxidative damage after SAH. Concomitant with the reduced neuroinflammation and oxidative stress, takinib improved functional outcome and neuronal survival after SAH. Mechanistically, takinib inhibited TAK1 activation and the subsequent ROS-NLRP3 inflammasome signaling pathway following SAH. In contrast, NLRP3 activation by nigericin abated the neuroprotective effects of takinib against EBI after SAH (**Figure 7**).

microglial polarization plays a critical role in the pathogenesis of neuroinflammation after SAH (29–31). Activated microglial exhibit different phenotypes under different stimulants and have distinct functions. M1-polarized microglial could increase proinflammatory cytokines release and aggravate ROS production. In contrast, M2-polarized microglial produce anti-inflammatory cytokines to keep the immune balance (29, 32). It has demonstrated that inhibition of M1 microglial polarization and induction microglial polarization into M2 could alleviate neuroinflammation and improve neurological outcomes after SAH (29). In addition, oxidative stress also participates in the development of EBI after SAH. Numerous studies have reported that ROS overproduction could induce neuroinflammation to exacerbate brain damage after SAH (1, 24, 33). Therefore, inhibition of M1-like microglial polarization and oxidative damage might be a successful strategy to reduce EBI after SAH.

Multiple studies have proved that TAK1 inhibition is a promising therapeutic application for various CNS diseases including traumatic brain injury (TBI), ischemic stroke, and SAH (34–36). For example, Shen et al. demonstrated that microglial-selective deletion of Tak1 inhibited IL-18 production and ameliorated ischemic stroke injury in prolonged obesity (37). Shi et al. reported that pharmacological inhibition of TAK1 with 5Z-7-oxozeaenol provided long-lasting improvement of stroke outcomes (38). In SAH area, TAK1 inhibition also attenuated EBI by reducing neuroinflammation and neuronal death (34). These indicated that TAK1 might be a promising target for treating SAH. However, the effects of TAK1 inhibition on microglial polarization and oxidative damage after SAH remain unclear.

It has been reported that 5Z-7-oxozeaenol is a potent selective inhibitor of TAK1 and vascular endothelial growth factor receptor 2 (39). Different with 5Z-7-oxozeaenol, takinib is a novel and highly selective TAK1 inhibitor. Recent studies have showed that takinib could effectively inhibit TAK1 activation-mediated inflammatory and apoptotic signaling cascades in different research fields (20, 21). However, to date, no study investigated the potential role of takinib on EBI after SAH. In our study, we first evaluated the effects of takinib on TAK1 expression after SAH. In accordance with previous studies, our data revealed that p-TAK1 (Thr 187) was significantly increased in neurons after SAH. In contrast, takinib treatment significantly decreased p-TAK1 expression. We then evaluated the possible influence of takinib on microglial polarization. Intriguingly, it showed that SAH insults significantly induced M1 microglial polarization, which could be inhibited by takinib treatment. Moreover, TAK1 inhibition by takinib increased M2 phenotype microglial. These suggested that TAK1 inhibition by takinib might have the potential to inhibit M1 microglial polarization and promote the microglial phenotype toward M2.

ROS overproduction plays an important role in the development of EBI after SAH. It can disrupt cellular functions by damaging nucleic acids, proteins, and lipids (1). Moreover, ROS production can aggravate neuroinflammation by activation of NLRP3 inflammasome signaling (25, 40). NLRP3 inflammasome has been demonstrated to participate in microglial polarization after SAH. Previous studies have demonstrated that inhibition of ROS/NLRP3 inflammasome signaling could decrease microglial M1 polarization and promote microglial polarization to M2 phenotype (29, 41). Our experiments revealed that TAK1 inhibition by takinib regulated microglial M1-M2 phenotype transition after SAH. However, the possible mechanisms remain elusive. Mounting evidence has showed that TAK1 activation could induce the aggravation of oxidative stress by promoting ROS production (16, 42). Inhibition of TAK1 is able to attenuate ROS overproduction and might be a potential therapeutic target for oxidative stress-related injuries. Interestingly, TAK1 has been verified as a key regulator of NLRP3 inflammasome activation (11, 34, 43). In our experiments, our data showed that takinib successfully inhibited the ROS overproduction and the subsequent activation of NLRP3 inflammasome signaling after SAH. Moreover, nigericin, a NLRP3 activator, abated the protective effects of takinib against EBI after SAH, validating the interaction between TAK1 and NLRP3 inflammasome. Concomitant with the reduced oxidative damage and neuroinflammation, takinib treatment significantly reduced neuronal apoptosis and improved functional behavior after SAH. Together with our experimental results, we provided the evidence that TAK1-ROS-NLRP3 inflammasome axis involved in the development of EBI after SAH. By intervening with TAK1-ROS-NLRP3 inflammasome axis, takinib modulated microglial polarization and inhibited oxidative damage.

It should be noted that NLRP3 inflammasome could modulate microglial polarization in a variety of disorders, including intracerebral hemorrhage, depression, ischemic stroke, white matter injury, as well as Alzheimer’s disease (44–46). These suggested that targeting NLRP3 inflammasome might be a feasible method to relieve neuroinflammation. In SAH area, Xu et al. reported that TAK1 inhibition by siRNA could reduce NLRP3 inflammasome-mediated neuronal pyroptosis (34). However, the previous studies did not investigate whether TAK1 inhibition affected microglial polarization. In the present study, we demonstrated that TAK1 could affect microglial polarization by modulating NLRP3 inflammasome. Inhibition TAK1 by takinib suppressed NLRP3 inflammasome and decreased M1 microglial polarization. However, how TAK1 regulates NLRP3 inflammasome is not fully investigated. In addition to ROS, K^+^ efflux, endosomal rupture, and mitochondrial dysfunction could trigger NLRP3 inflammasome activation (47). Hindi et al. indicated that TAK1 plays a critical role in regulating skeletal muscle mass and oxidative metabolism. TAK1 activation could induce an accumulation of dysfunctional mitochondria as well as oxidative damage in skeletal muscle (19). We suspected that TAK1 might also affect mitochondrial dysfunction to trigger NLRP3 inflammation. But further studies are still needed to decipher this question and whether other molecular targets are involved in this modulation.

Our study has several limitations. Firstly, the long-term effects of TAK1 inhibition in the delayed phase of SAH remains unclear. Meanwhile, no studies have performed to investigate the influence of TAK1 on cerebral vasospasm and delayed ischemic neurologic deficits after SAH. A recent study by Shen et al. indicated that prolonged high-fat diet (for 32 weeks) feeding-induced obesity has a significant cerebrovascular dysfunction, which is closely associated with microglial TAK1 activation. They showed that microglial TAK1 activation significantly aggravated basilar artery abnormalities. Both pharmacological suppression and genetic microglial-selective TAK1 deletion relieved basilar artery dysfunction and improved the outcome of ischemic stroke (37). On this background, we suspected that TAK1 inhibition might ameliorate cerebral vasospasm and relieve delayed ischemic neurologic deficits in the late phase of SAH. Secondly, the intracerebroventricular administration limits the clinical utility of takinib. Other administration routes instead of ventricle injections should be conducted to validate its clinical translatability. Thirdly, our data indicated that takinib could protect against M1 microglial polarization and oxidative damage after SAH by modulation of TAK1-ROS-NLRP3 inflammasome axis. However, whether these effects are mediated by NF‐κB, AMPK, and p38 should be further determined (48, 49). Lastly, in addition to activate NLRP3, nigericin might modulate other signaling targets to aggravate neuroinflammation. Thus, more experiments are needed to solve these questions.

## 5 Conclusion

In conclusion, we provided the first evidence that takinib could modulate microglial polarization and inhibit oxidative damage after SAH primarily by targeting the TAK1-ROS-NLRP3 inflammasome axis. These findings implied that takinib might be a potential new therapeutic candidate for the treatment of SAH.

## Data availability statement

The original contributions presented in the study are included in the article/**Supplementary Material**. Further inquiries can be directed to the corresponding authors.

## Ethics statement

The animal study was approved by the Animal Ethics Review Committee of Beijing Friendship Hospital. The study was conducted in accordance with the local legislation and institutional requirements.

## Author contributions

WW: Conceptualization, Formal analysis, Investigation, Methodology, Project administration, Writing – original draft, Writing – review & editing. CP: Formal analysis, Investigation, Methodology, Project administration, Visualization, Writing – original draft. JZ: Investigation, Methodology, Validation, Writing – review & editing. LP: Methodology, Writing – review & editing. XZ: Methodology, Supervision, Writing – review & editing. LS: Conceptualization, Methodology, Project administration, Writing – original draft, Writing – review & editing. HZ: Conceptualization, Formal analysis, Funding acquisition, Project administration, Supervision, Writing – review & editing.

## Supplementary material

Supplementary Figure 1 Schematic illustration of experiment design.
