# Supplementary figures and images for: Takinib inhibits microglial M1 polarization and oxidative damage after subarachnoid hemorrhage by targeting TAK1-dependent NLRP3 inflammasome signaling pathway
Source: Front Immunol. 2023 Nov 14;14:1266315. doi: 10.3389/fimmu.2023.1266315 (PMC10682771; doi:10.3389/fimmu.2023.1266315)

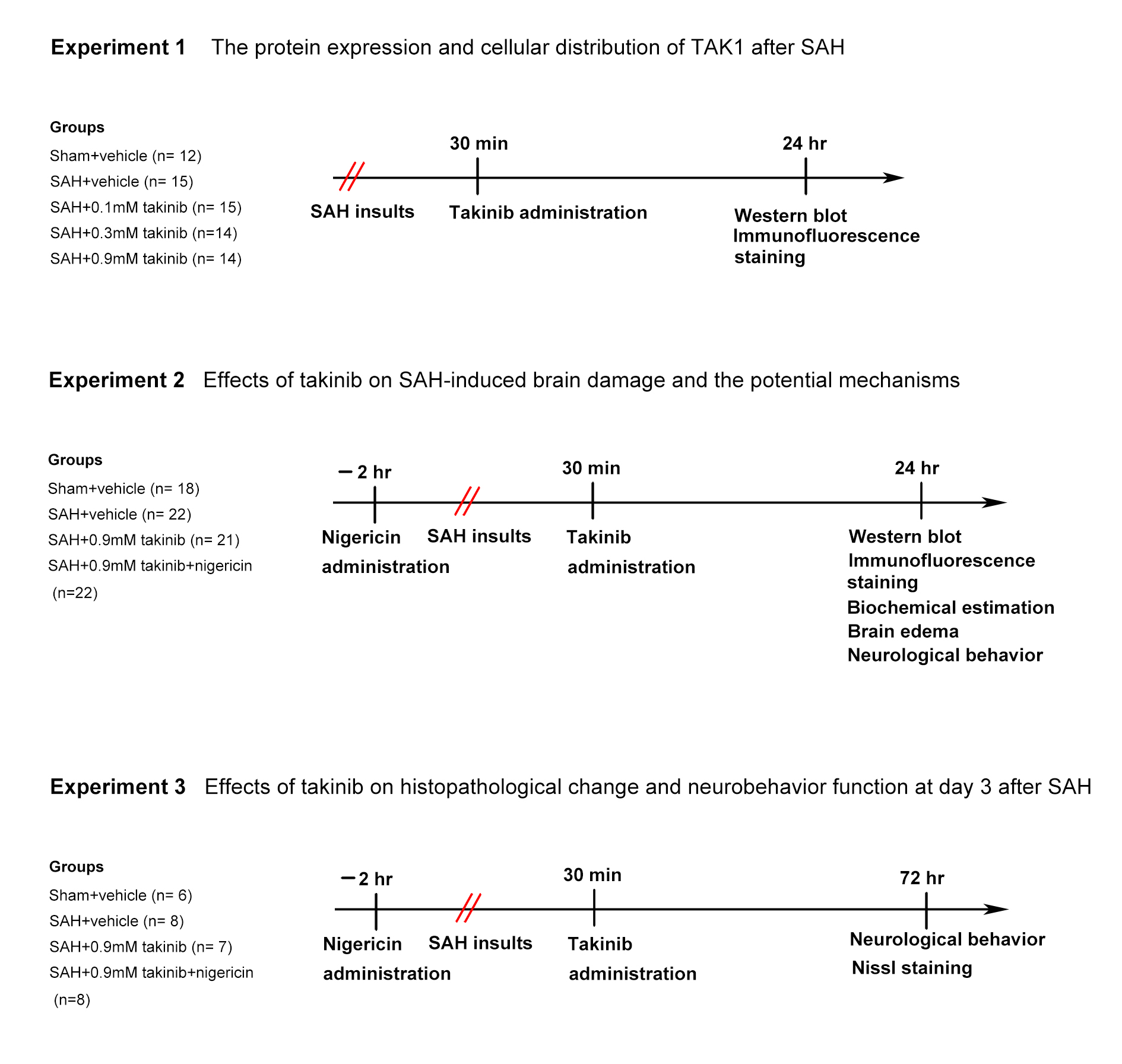

Supplement: Supplementary Figure 1 — Schematic illustration of experiment design. [file Image_1.jpeg]
